# Application and potential value of curcumin in prostate cancer: a meta-analysis based on animal models

**DOI:** 10.3389/fphar.2024.1379389

**Published:** 2024-05-09

**Authors:** Shiheng Wang, Fengxia Zhang, Jing Chen

**Affiliations:** ^1^ College of Medical Imaging Laboratory and Rehabilitation, Xiangnan University, Chenzhou, China; ^2^ Institute for History of Medicine and Medical Literature, China Academy of Chinese Medical Sciences, Beijing, China

**Keywords:** curcumin, prostate cancer, animal models, systematic review, meta-analysis

## Abstract

**Introduction:**

Curcumin is gaining recognition as an agent for cancer chemoprevention and is presently administered to humans. However, the limited number of clinical trials conducted for the treatment of prostate cancer is noteworthy. Animal models serve as valuable tools for enhancing our understanding of disease mechanisms and etiology in humans. The objective of this study was to examine the anti-prostate cancer effects of curcumin *in vivo* for comprehending its current research status and potential clinical applicability.

**Methods:**

Our methodology involved a systematic exploration of animal studies pertaining to curcumin and prostate cancer, as documented in PubMed, Web of Science, Embase, Cochrane Library, CNKI, Wanfang database, Vip database, and SinoMed, up to 03 September 2023. Risk of bias was assessed using the SYRCLE Animal Study Risk of Bias tool. The results were combined using the RevMan 5.3.

**Results:**

A comprehensive analysis was conducted on 17 studies encompassing 263 mouse transplantation tumor models. The findings of this meta-analysis demonstrated that curcumin exhibited a superior inhibitory effect on the volume of prostate cancer tumors in mice compared to the control group (standardized mean difference [SMD]: 1.16, 95% confidence interval [CI]: 0.52, 1.80, *p* < 0.001). Additionally, curcumin displayed a more effective inhibition of mice prostate cancer tumor weight (SMD: −3.27, 95% CI: −4.70, −1.83, *p* < 0.001). Furthermore, in terms of tumor inhibition rate, curcumin exhibited greater efficacy (SMD: 0.25, 95% CI: 0.23, 0.27, *p* < 0.001). Moreover, curcumin more effectively inhibited PCNA mRNA (SMD: −3.11, 95% CI: −4.60, −1.63, *p* < 0.001) and MMP2 mRNA (SMD: −3.19, 95% CI: 5.85, −0.53, *p* < 0.001).

**Conclusion:**

Curcumin exhibited inhibitory properties towards prostate tumor growth and demonstrated a beneficial effect on prostate cancer treatment, thereby offering substantiation for further clinical investigations. It is important to acknowledge that the included animal studies exhibited considerable heterogeneity, primarily because of the limited number of studies included. Consequently, additional randomized controlled trials are required to comprehensively assess the efficacy of curcumin in humans.

**Systematic Review Registration:**

(https://www.crd.york.ac.uk/prospero/display_record.php?ID=CRD42023464661), identifier (CRD42023464661).

## 1 Introduction

Prostate cancer (PCa) is a malignant neoplasm associated with high morbidity and mortality rates. Statistical data indicate that in 2020, there were a projected total of 1,414,259 newly diagnosed cases of PCa worldwide, constituting approximately 7.3% of all newly diagnosed patients with cancer and positioning it as the second most prevalent cancer type. Additionally, PCa is expected to cause 375,304 deaths by, accounting for approximately 3.8% of all cancer-related fatalities (Sung et al., 2021). The prevalence and fatality rates of PCa have increased substantially within the framework of the progressively aging population. Projections indicate that by 2040, the global incidence of PCa is anticipated to surge to approximately 2.3 million fresh cases, accompanied by 740,000 fatalities (National Health Commission of the People’s Republic of China, 2019; Ferlay et al.). PCa has emerged as a significant determinant of wellbeing and mortality in men. The principal therapeutic modalities for PCa include surgery, radiotherapy, and chemotherapy. Nevertheless, these interventions are not without limitations and may adversely affect patients’ quality of life; hence, it is imperative to address the constraints of existing therapies by advancing and implementing novel anticancer drugs that exhibit enhanced therapeutic efficacy and minimize adverse effects. In this context, herbal extracts have garnered considerable attention, as substantiated by scientific research indicating their potential to inhibit tumor growth (Singh et al., 2022; Bai et al., 2023). Curcumin, a polyphenol derived from turmeric, is commonly used in culinary applications owing to its antioxidant, antimicrobial, and anti-inflammatory properties (Hewlings and Kalman, 2017). A previous meta-analysis has demonstrated the therapeutic potential of curcumin for the treatment of malignant tumors (de Oliveira et al., 2022). To date, there is a lack of published meta-analyses examining the efficacy of curcumin in PCa treatment. Nonetheless, curcumin has demonstrated a significant effect on various PCa cell types when used as a therapeutic intervention (Termini et al., 2020). Previous studies have demonstrated that curcumin has multiple mechanisms of action in relation to PCa. Primarily, it exerts inhibitory effects on the prostate-specific antigen (PSA) by reducing its function and inhibiting its activity, thereby leading to decreased transcriptional activity of the androgen receptor (AR) and diminished expression of AR protein in LNCaP cells (Hewlings and Kalman, 2017). Furthermore, it has been demonstrated that curcumin can impede the activation of NF-κB induced by tumor necrosis factor and facilitate apoptosis in cells affected by PCa (Yang et al., 2005). Additionally, curcumin can hinder PCa progression by upregulating miR-143 and FOXD3 in DU145 and PC-3 cells and concurrently downregulating PGK1 expression (Cao et al., 2017). Furthermore, the inhibition of metastasis and survival of DU145 and PC-3 cells by curcumin *via* the Notch-1 signaling pathway has garnered significant interest in the medical field, highlighting the potential therapeutic value of curcumin in the management of PCa.

Despite the existence of prospective randomized controlled trials on curcumin for PCa (Ide et al., 2010; Hejazi et al., 2016), the limited number of studies and the lack of uniform outcome indicators prevents the execution of a meta-analysis. Currently, there is a growing body of research focusing on animal testing of curcumin for PCa, as animal models serve as valuable tools to enhance our understanding of human disease mechanisms and etiology (Sena et al., 2014). Understanding the mechanisms underlying the therapeutic effects of curcumin in PCa remains incomplete, indicating the need for further investigation. Consequently, the clinical use of curcumin remains a distant prospect. Moreover, the translation of findings from animal studies into human clinical trials poses significant challenges. In this regard, meta-analyses of animal study data are valuable as they facilitate the identification of disparities between preclinical and clinical trial outcomes and aid in enhancing the design of clinical trials (Vesterinen et al., 2014). The primary and paramount recommendation to enhance reproducibility and translation, as outlined by Spanagel, is to perform preclinical meta-analyses (Spanagel, 2022). Hence, our objective was to investigate the therapeutic effects and potential significance of curcumin in animal models of PCa to offer a point of reference for clinical investigation and pharmaceutical advancement.

## 2 Data and methods

### 2.1 Registration

This study adhered to the PRISMA guidelines for reporting systematic reviews and was prospectively registered with PROSPERO (https://www.crd.york.ac.uk/PROSPERO/#myprospero) (ID: CRD42023464661).

### 2.2 Search strategy

PubMed, Embase, Cochrane Library, Web of Science, CNKI, Wanfang, VIP, and SinoMed databases were systematically searched from their inception until 03 September 2023. In addition, a manual search of the references of the included studies was performed. The search strategy was designed based on the following criteria: (1) study population: animal models of PCa and (2) intervention: curcumin. Table 1 presents the search strategy used for the PubMed database.

**TABLE 1:** PubMed search strategy.

Search number	Query
1	Curcumin [MeSH Terms]
2	(((((((Curcumin [Title/Abstract]) OR (1,6-Heptadiene-3,5-dione, 1,7-bis(4-hydroxy-3-methoxyphenyl)-, (E,E)-[Title/Abstract])) OR (Turmeric Yellow [Title/Abstract])) OR (Yellow, Turmeric [Title/Abstract])) OR (Curcumin Phytosome [Title/Abstract])) OR (Phytosome, Curcumin [ Title/Abstract])) OR (Diferuloylmethane [Title/Abstract])) OR (Mervia [Title/Abstract]))
3	1 OR 2
4	Prostatic Neoplasms OR Prostatic Intraepithelial Neoplasia [MeSH Terms]
5	1. ((prostat* [Title/Abstract] AND (cancer* [Title/Abstract] OR malignan* OR carcinom* [Title/Abstract] OR tumo* OR neopla* [Title/Abstract] OR adenocarcinom* OR intraepithelial OR adeno* [Title/Abstract])
6	4 OR 5
7	3 AND 6

### 2.3 Inclusion criteria

#### 2.3.1 Research object

(1) Animal models were limited to rats and mice; (2) PCa models; (3) complete papers, not abstracts; (5) the resulting data were available and could be extracted; (6) control group; and (7) no restrictions on publication time and language.

#### 2.3.2 Intervention

(1) The experimental group was only administered a certain dose of curcumin, and the source of curcumin was not restricted; (2) the control group was administered equal amounts of placebo (such as normal saline and polyethylene glycol) or other drugs (such as paclitaxel and alpha-tomatine); and (3) there was no restriction on the method of taking the medicine, either orally or by injection.

#### 2.3.3 Outcome measures

Tumor volume (calculated using the formula: Tumor volume (mm3) = π/6 (long diameter × short diameter)2), tumor weight (g), tumor inhibition rate (calculated as (mean tumor weight of control group - mean tumor weight of experimental group)/mean tumor weight of control group * 100%), proliferating cell nuclear antigen-mRNA (PCNA-mRNA), and matrix metallo peptidase (matrix metallo peptidase2-mRNA, MMP2-mRNA). (5) Type of study: animal experiment (randomized control: intervention and control groups). There were no restrictions on the publication time or language.

### 2.4 Exclusion criteria

(1) Studies with incomplete or unanalyzable data; (2) cell tests, reviews, abstracts, letters, plans, or *in vitro* studies; (3) curcumin was used in the control group; and (4) curcumin was not the main component of the intervention in the experimental group.

### 2.5 Literature screening

Two researchers (Wang and Zhang) independently used the literature management software EndnoteX9 to screen literature. First, duplicate literature is excluded by searching for duplicate literature. Titles and abstracts were read according to the inclusion and exclusion criteria. If the PICOS met the inclusion criteria, the study was included. Documents were excluded if the exclusion criteria were met. Finally, we read the full text and judge whether the literature meets the inclusion criteria, and if it does, we include it. After screening was completed, the two participants were compared, and if the results were the same, the included study was finally determined. Otherwise, a third researcher (Jing Chen) was consulted or the decision was discussed.

### 2.6 Data extraction

Two researchers (Wang and Zhang) designed the data extraction tables based on the information required for the research. After reaching a mutual agreement, the necessary information was filtered. Two studies independently extracted data from articles or graphs. The extracted information included the first author, year, sample size, age, intervention, study duration, dose, and outcome indicators. If the data could not be extracted, the author was contacted via email for missing or additional data. Finally, the extracted data were entered into EXCEL. If the extracted outcome index data were continuous, they were uniformly expressed as means and SD. If the outcome index data were binary, they were expressed as the number of occurrence cases ^®^ and number of samples (n). If not, they were converted. If the information extracted by the two people was inconsistent, it was first discussed upon. On still being unsolved, it was either further discussed upon or a third researcher was consulted to decide (Chen).

### 2.7 Quality evaluation

Two researchers (Wang and Zhang) used the SYRCLE Animal Studies Bias Risk Tool (Hooijmans et al., 2014a) to conduct bias risk assessment. SYRCLE’s Risk of Bias tool is an adapted version of the Cochrane risk-of-bias tool used in animal intervention studies. This was an objective assessment of possible biases or confusion in the tools used for the design, conduct, and measurement of the animal experiments. Its suitability for different types and domains of animal experiments covered six areas of bias: selection, implementation, measurement, loss to follow-up, reporting, and others. In each area, the risk of bias was judged to be low (+), unclear (?), or high risk (−). Two researchers independently assessed the risk of bias in the included studies, and if there was a disagreement, a third researcher (Chen) was consulted for resolution.

### 2.8 Statistical analysis

The meta-analysis was conducted using Revman5.4 and Stata 16.0 software. Relative risk (RR) was employed for dichotomous data, whereas standardized mean difference (SMD) was used for continuous data. The effect estimates were calculated using 95% confidence intervals (CIs). Initially, a heterogeneity test was conducted to address anticipated heterogeneity, with the I2 statistic (Higgins and Thompson, 2002) being employed for this purpose. In light of the absence of uniformity across animal studies, a random-effects model was chosen, and various statistical techniques, such as meta-regression, subgroup analyses, and sensitivity analyses, were employed to investigate the origins of heterogeneity. Funnel plots were generated to examine publication bias, adhering to the Cochrane Handbook’s recommendation to include at least 10 relevant literature sources. Assessment of publication bias involved a visual assessment of funnel plot asymmetry.

## 3 Results

### 3.1 Results of literature screening

**FIGURE 1:**
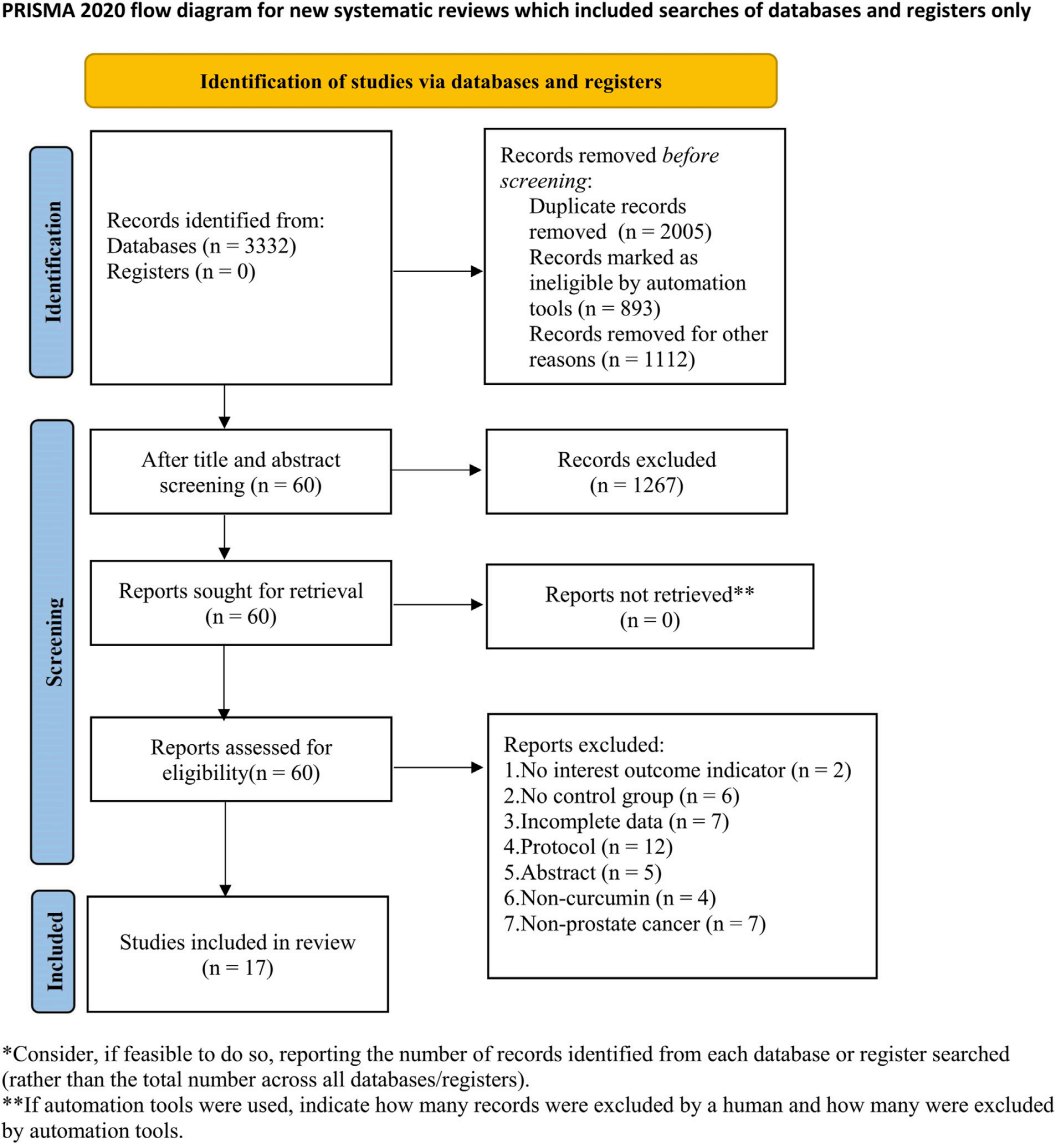
Literature screening process.

A comprehensive search was conducted on 3,332 papers, eliminating 2,005 duplicates. Subsequently, 1,267 papers were excluded based on an evaluation of their titles and abstracts. Upon further examination of the full text, an additional 43 papers were excluded for various reasons, such as the absence of endpoint indicators suitable for inclusion, lack of a control group, incomplete data, inadequate trial plan or abstract, intervention not involving curcumin, and absence of PCa. Ultimately, 17 animal trials were deemed eligible for inclusion. Figure 1 shows the results of the literature review.

### 3.2 Basic characteristics

Seventeen animal experiments involving the use of mouse transplantation tumor models were included in the analysis. The experimental group consisted of 132 mice, whereas the control group consisted of 131 mice, resulting in a total of 263 mice used in the study. Among the included studies, 13 originated in China (Limin et al., 2007; Qi, 2009; ZHAO et al., 2010; LI et al., 2013; Zhang, 2013; Huang et al., 2015; QI et al., 2015; Yang et al., 2015; Ye, 2015; Roy et al., 2016; Zhao et al., 2018; MAO et al., 2019; Cheng et al., 2020). Additionally, three studies were conducted in the United States (Dorai et al., 2001; Khor et al., 2006; Barve et al., 2008), while one study was conducted in Spain (Fernández-Martínez et al., 2009). Curcumin, obtained from Sigma (USA), was administered to the test group predominantly intraperitoneally, with only a few studies utilizing oral administration. The control group, in contrast, received a placebo intervention. Table 2 summarizes the basic characteristics of the included studies.

**TABLE 2:** Basic characteristics of the included studies.

First author	Country	Year	Age	Cell types	Intervention (I/C)	Administration	Cases (I/C)	Treatment (week)	Outcome indicator
Zhang (2013)	China	2013	4w	PC-3	Curcumin placebo	intraperitoneal injection	6.6	4	Tumor volume, tumor weight
Ye (2015)	China	2015	4-5w	PC-3	Curcumin, Polyethylene glycol	intraperitoneal injection	5.7	2	Tumor volume, PCNAmRNA
QI et al. (2015)	China	2015	4w	PC-3	Curcumin, saline	intraperitoneal injection	6.6	4	MMP2mRNA
Qi (2009)	China	2009	4w	PC-3	Curcumin, Paclitaxel	intraperitoneal injection	6.6	4	Tumor suppressor, PCNAmRNA, MMP2 mRNA
MAO et al. (2019)	China	2019	4-5w	RM-1	Curcumin, DMSO	intraperitoneal injection	10.10	2	Tumor weight, tumor suppression
Limin et al. (2007)	China	2007	4-6w	PC-3	Curcumin, polyethylene glycol	intraperitoneal injection	6.6	4	Tumor volume, tumor weight, tumor inhibition
Roy et al. (2016)	China	2016	4w	PC-3	Curcumin, Polyethylene glycol	intraperitoneal injection	6.6	4	tumor weight
LI et al. (2013)	China	2013	4-6w	PC-3	Curcumin, saline	intraperitoneal injection	12.12	4	Tumor volume, tumor weight, tumor inhibition
ZHAO et al. (2010)	China	2010	4-6w	PC-3	Curcumin, placebo	intraperitoneal injection	6.6	4	Tumor volume, PCNAmRNA,MMP2mRNA
Zhao et al. (2018)	China	2017	-	LNCaP	Curcumin, DMSO	intraperitoneal injection	4.4	7	Tumor volume
Yang et al. (2015)	China	2015	-	PC-3	Curcumin, Polyethylene glycol	intraperitoneal injection	8.8	4	Tumor volume, tumor weight, tumor inhibition
Dorai et al. (2001)	United States of America	2001	6-8w	LNCaP	Curcumin, feed	profess conviction	10.10	6	Tumor volume
Khor et al. (2006)	United States of America	2006	6w	PC-3	Curcumin, PEITC	intraperitoneal injection	12.12	4	Tumor volume, tumor weight
Huang et al. (2015)	China	2015	6-7w	PC-3	Curcumin, α-Tomatine	intraperitoneal injection	9.9	4	Tumor volume, tumor weight
Fernández-Martínez et al. (2009)	Spain	2009	5-6w	PC-3	Curcumin, NS-398	intraperitoneal injection	8.8	4	Tumor volume, tumor weight, MMP2mRNA
Cheng et al. (2020)	China	2020	5-8w	LC540	Curcumin, DMSO	intraperitoneal injection	6.6	1	Tumor volume, tumor weight
Barve et al. (2008)	United States of America	2008	-	PC-3	Curcumin, PEITC	profess conviction	12.9	16	PCNAmRNA

I, test group; C, control group; DMSO, dimethyl sulfoxide; PEITC, phenethyl isothiocyanate.

### 3.3 Risk of bias assessment

**FIGURE 2:**
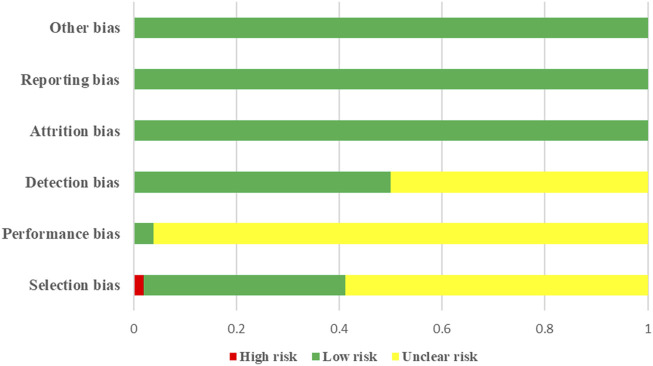
Results of risk of bias assessment.

**FIGURE 3:**
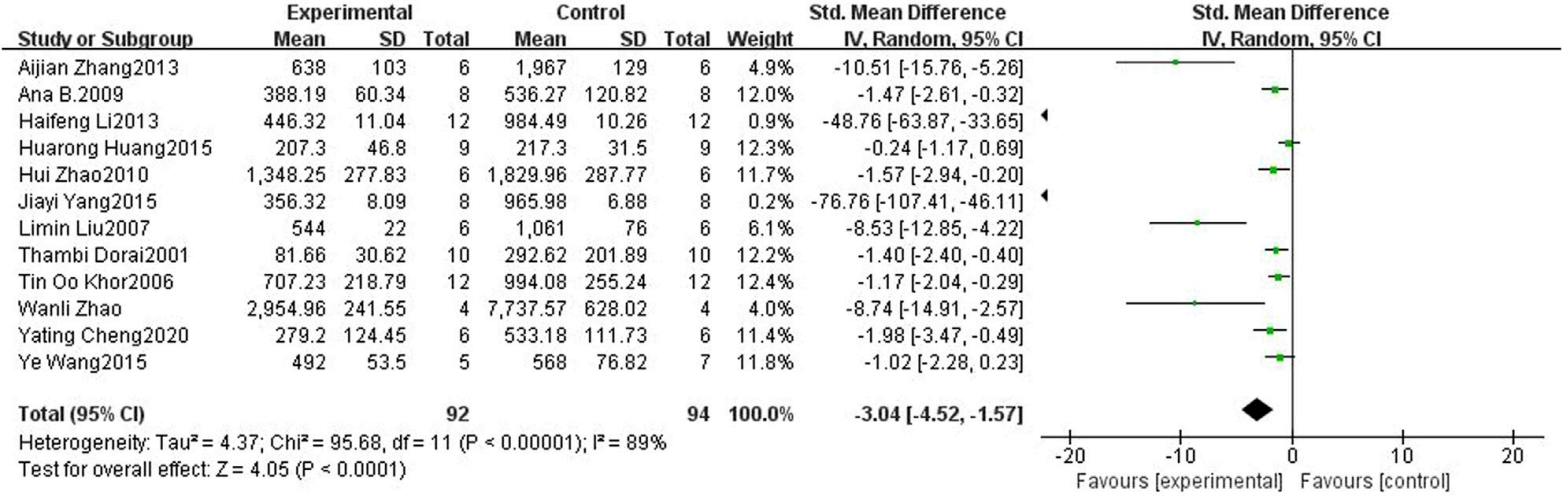
Forest plot of tumor volume for curcumin treatment of prostate cancer.

Three studies (QI et al., 2015; Roy et al., 2016; MAO et al., 2019) used the random number table method to address selection bias, whereas one study (Dorai et al., 2001) employed randomization to allocate participants into intervention groups. The remaining studies did not provide sufficient information on their approaches to selection bias. Regarding implementation bias, only one study (ZHAO et al., 2010) reported the use of randomization and blinding, whereas the remaining studies did not explicitly state their methods. Blinding was not described in any of the studies, indicating potential measurement bias. The risks of lost visits, reporting, and other biases were relatively low. Figures 2, 3 show the results of the risk of bias assessment.

### 3.4 Meta-analysis results

#### 3.4.1 Tumor volume

**FIGURE 4:**
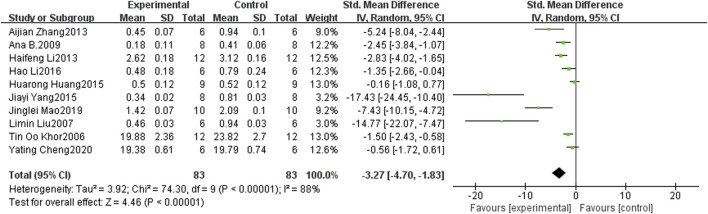
Forest plot of tumor weight for curcumin treatment of prostate cancer.

Twelve studies (Dorai et al., 2001; Higgins and Thompson, 2002; Khor et al., 2006; Limin et al., 2007; Fernández-Martínez et al., 2009; ZHAO et al., 2010; LI et al., 2013; Zhang, 2013; Hooijmans et al., 2014a; Huang et al., 2015; Yang et al., 2015; Ye, 2015; Roy et al., 2016; Zhao et al., 2018; Cheng et al., 2020) included in the analysis provided data on the tumor volume. The findings revealed a noteworthy suppressive effect of curcumin on PCa tumor volume in mice compared to that in the control groups (SMD: 1.16, 95% CI: 0.52, 1.80, *p* < 0.001). A considerable heterogeneity was observed among the included studies (I2 = 89%). Figure 4 shows a forest plot of tumor volume after curcumin treatment of PCa.

#### 3.4.2 Tumor weight

**FIGURE 5:**
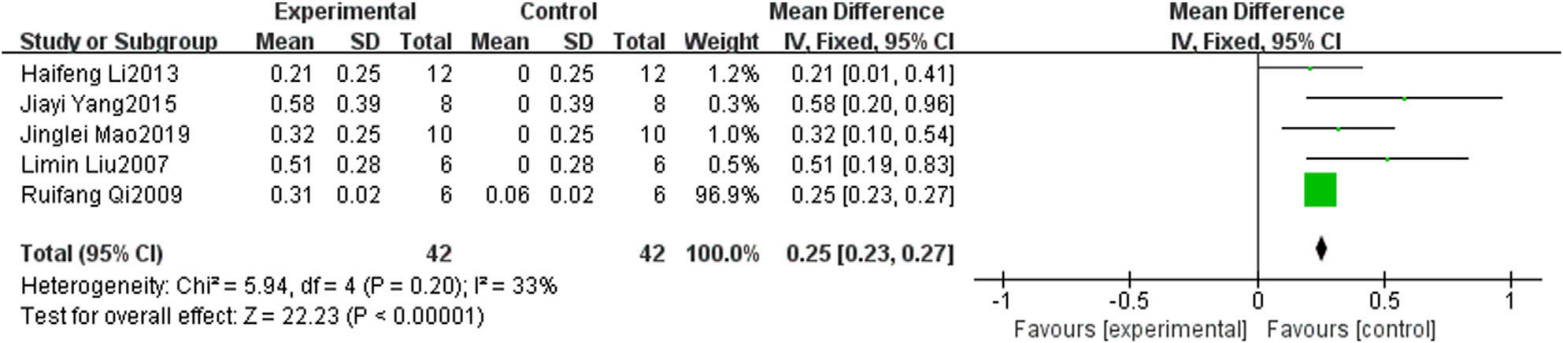
Forest plot of tumor inhibition rate for curcumin treatment of prostate cancer.

Ten studies (Khor et al., 2006; Limin et al., 2007; Fernández-Martínez et al., 2009; LI et al., 2013; Zhang, 2013; Huang et al., 2015; Yang et al., 2015; Roy et al., 2016; Cheng et al., 2020) provided data on tumor weight, and the findings revealed a significant inhibitory effect of curcumin on the tumor weight of PCa in mice when compared to the control groups (SMD: −3.27, 95% CI: −4.70, −1.83, *p* < 0.001). Notably, substantial heterogeneity was observed among the included studies (I2 = 88%). Figure 5 shows a forest plot of tumor weight after curcumin treatment of PCa.

#### 3.4.3 Tumor suppression rate

**FIGURE 6:**
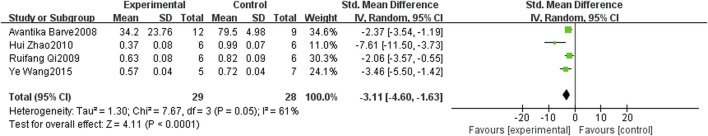
Forest plot of PCNA mRNA for curcumin treatment of prostate cancer.

Tumor suppression rates were reported in five studies (Limin et al., 2007; Qi, 2009; LI et al., 2013; Yang et al., 2015; MAO et al., 2019). The findings indicated that curcumin exhibited a significantly higher tumor inhibition rate in mouse PCa than in the control group (SMD: 0.90, 95% CI: 0.44, 1.36, *p* < 0.001). Figure 6 shows a forest plot of the tumor inhibition rate of curcumin treatment in PCa.

#### 3.4.4 PCNA mRNA

**FIGURE 7:**
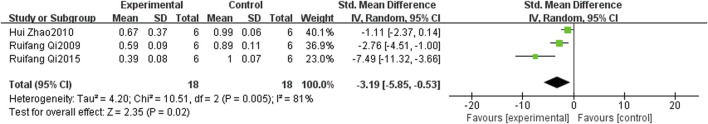
Forest plot of MMP2 mRNA for curcumin treatment of prostate cancer.

Four studies (Barve et al., 2008; Qi, 2009; ZHAO et al., 2010; Ye, 2015) documented the presence of PCNA mRNA. The findings indicated that curcumin exhibited superior inhibition of PCNA mRNA in mice PCa tumors when compared to the control group (SMD: −3.11, 95% CI: −4.60, −1.63, *p* < 0.001). Figure 7 shows a forest plot of PCNA mRNA expression after curcumin treatment in PCa.

#### 3.4.5 MMP2 mRNA

**FIGURE 8:**
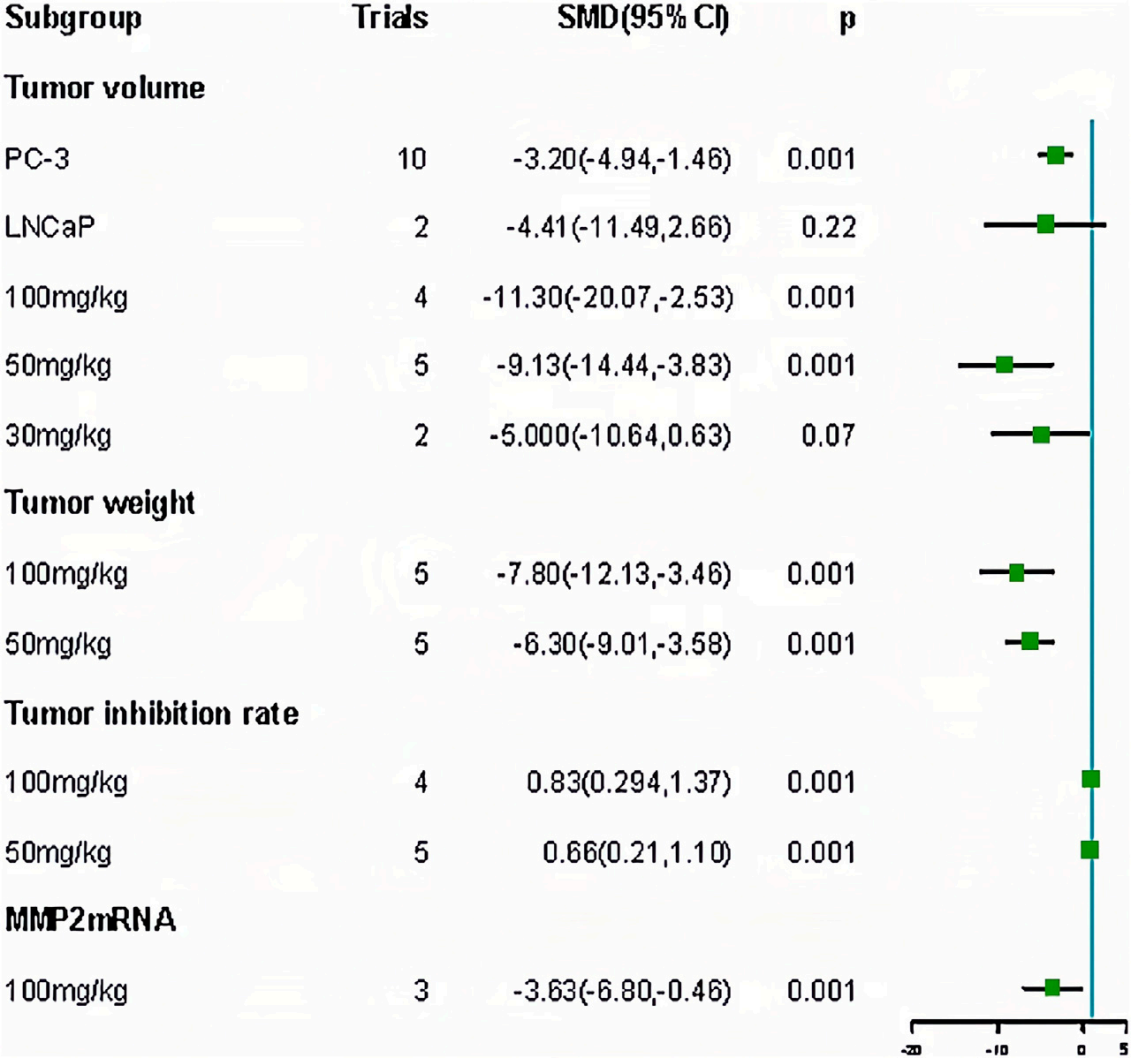
Forest plot of subgroup for curcumin treatment of prostate cancer.

Three studies (Qi, 2009; ZHAO et al., 2010; QI et al., 2015) examined the expression of MMP2 mRNA and found that curcumin demonstrated a significantly greater inhibitory effect on MMP2 mRNA in a mice transplant tumor model of PCa when compared to the control group (SMD: −3.19, 95% CI: −5.85, −0.53, *p* < 0.001). Figure 8 shows a forest plot of MMP2 mRNA expression after curcumin treatment in PCa.

### 3.5 Meta regression

We performed a meta-analysis of duration and dose. The results showed that the curcumin dose affected the tumor inhibition rate (*p* < 0.05) (Table 3). Subsequently, we performed a subgroup analysis of the doses. Subgroup analysis showed that the effect of 100 mg/kg curcumin (SMD: 0.83, 95% CI: 0.294.1.37) was better than 50 mg/kg curcumin (SMD: 0.66, 95% CI: 0.21.1.10), which proved that the meta regression results were correct (Table 4).

**TABLE 3:** Meta-regression results of curcumin in the treatment of prostate cancer.

Intervention	Trials	Outcome indicator	
		Treatments	Dosages
MMP2 mRNA	3	-	0.603
PCNAmRNA	4	0.557	0.808
Tumor volume	12	0.071	0.509
tumor weight	10	0.658	0.183
Tumor Suppression Rate	5	0.286	0.033

**TABLE 4:** Results of subgroup analysis.

Outcomes	Subgroup	Trials	SMD95% CI	P
Tumor volume	PC-3	10	−3.20 (-4.94,-1.46)	0.0001
	LNCaP	2	−4.41 (−11.49.2.66)	0.22
	100 mg/kg	4	−11.30 (-20.07,-2.53)	0.0001
	50 mg/kg	5	−9.13 (-14.44,-3.83)	0.0001
	30 mg/kg	2	−5.000 (−10.64.0.63)	0.069
Tumor weight	100 mg/kg	5	−7.80 (-12.13,-3.46)	0.0001
	50 mg/kg	5	−6.30 (-9.01,-3.58)	0.0001
Tumor inhibition rate	100 mg/kg	4	0.83 (0.294.1.37)	0.0001
	50 mg/kg	5	0.66 (0.21.1.10)	0.0001
MMP2mRNA	100 mg/kg	3	−3.63 (-6.80,-0.46)	0.0001

### 3.6 Subgroup and sensitivity analyses

**FIGURE 9:**
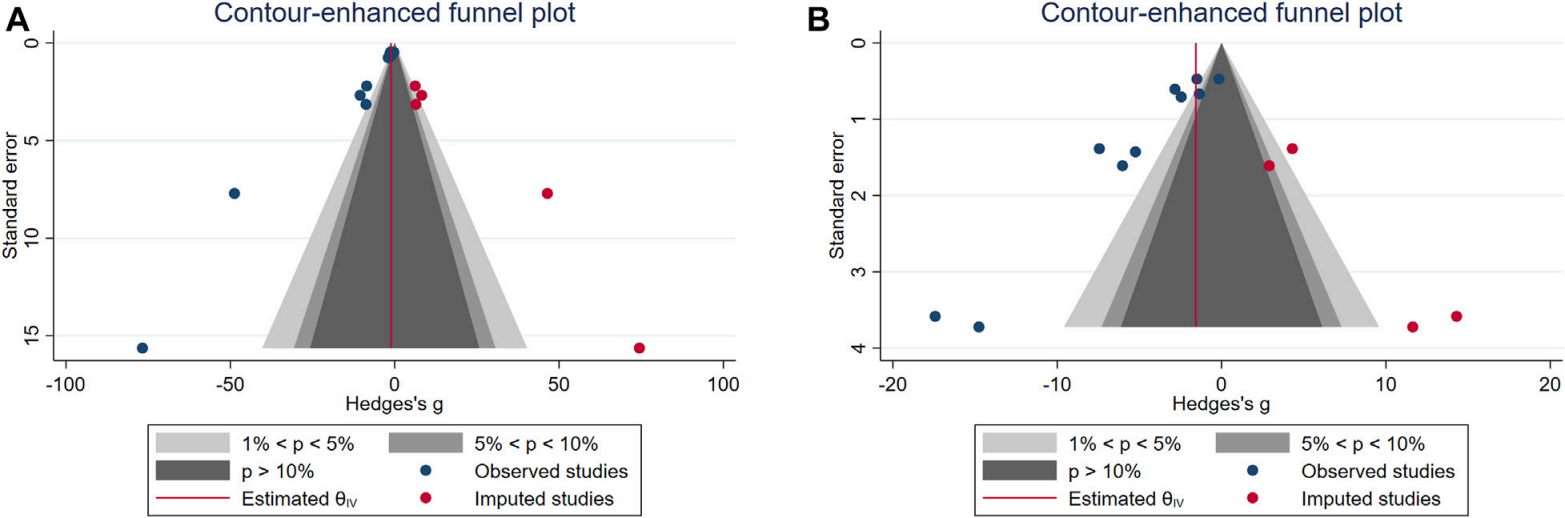
**(A)** Weighted funnel plot of tumor volume. **(B)** Weighted funnel plot of tumor weight.

We performed a subgroup analysis of PCa cell types and doses. The results showed that: (1) ginger had different effects on different PCa cells. The effect of the PC - 3 (SMD: 11.30, 95% CI: 20.07, 2.53) was superior to that of the LNCaP (SMD: 4.41, 95% CI: 11.49, 2.66). The effect of the PC - 3 (SMD: 11.30, 95% CI: 20.07, 2.53) was superior to the LNCaP (SMD: 4.41, 95% CI: 11.49, 2.66). (2) Different doses of curcumin had different effects on outcome indexes. In terms of tumor volume, effect of ranking was 100 mg/kg (SMD: 11.30, 95% CI: 20.07, 2.53) > 50 mg/kg (SMD: 9.13, 95% CI: 14.44, 3.83) > 30 mg/kg (SMD: 5.00, 95% CI: 10.64, 0.63). The effect on tumor weight was 100 mg/kg (SMD: 7.80, 95% CI: 12.13, 3.46) > 50 mg/kg (SMD: 6.30, 95% CI: 9.01, 3.58). The tumor inhibition rate was 100 mg/kg (SMD: 0.83, 95% CI: 0.294, 1.37) to 50 mg/kg (SMD: 0.66, 95% CI: 0.21, 1.10). The results of the subgroup analysis showed a positive correlation between the curcumin dose and results; the higher the dose, the better the effect (Table 4; Figure 9). Contour-weighted funnel plots and cut-and-complement methods showed a low likelihood of publication bias (Figure 9, Table 5).

**TABLE 5:** Results of publication bias detection.

Outcomes	Trim and fill method outcome	Hedges’s g	95% CI
Tumor volume	Observed	−1.34	−1.75,-0.94
	Observed + Imputed	−1.144	−1.54,-0.73
Tumor weight	Observed	−1.96	−2.43,-1.493
	Observed + Imputed	−1.56	−2.018,-1.10

Sensitivity analysis was performed on the results of the meta-analysis. The following results were obtained: tumor volume (SMD: −3.04, 95% CI: 4.51,-1.57, *p* = 0.0001), tumor weight (SMD: 3.62, 95% CI: 5.14,-2.09, *p* = 0.0001), tumor inhibition rate (SMD: 0.90, 95% CI: 0.44, 1.36, *p* = 0.0001), PCNA mRNA (SMD: 3.45, 95% CI: 5.10, 1.81, *p* = 0.0001), and MMP2 mRNA (SMD: 3.56, 95% CI: 6.46, 0.67, *p* = 0.0001). The results of sensitivity analysis showed that the results of the meta-analysis were stable (Table 6).

**TABLE 6:** Sensitivity analysis results.

Outcomes	SMD95% CI	P
Tumor volume	−3.04 (−4.51,-1.57)	0.0001
Tumor weight	−3.62 (-5.14,-2.09)	0.0001
Tumor Suppression Rate	0.90 (0.44.1.36)	0.0001
PCNAmRNA	−3.45 (−5.10,-1.81)	0.0001
MMP2mRNA	−3.56 (-6.46,-0.67)	0.0001

## 4 Discussion

### 4.1 Summary of evidence

This study included 17 meta-analyses that aimed to examine the evidence derived from animal studies on the therapeutic efficacy of curcumin in the treatment of PCa. The findings indicated that curcumin exhibited inhibitory effects on PCNA and MMP2 mRNA expression and the growth of malignant tumors in PCa cells of animal models when compared to control groups. The stability of the results was confirmed through a sensitivity analysis. Furthermore, the meta-regression analysis demonstrated that the dosage of curcumin had a significant impact on the rate of tumor inhibition in the animal model. Through subgroup analysis, a positive correlation between the dosage of curcumin and the outcomes was determined, indicating that higher doses of curcumin yielded more favorable effects.

### 4.2 Mechanisms underlying the therapeutic effects of curcumin in PCa management

Curcumin exhibits anticancer properties, as demonstrated in a meta-analysis conducted by de Oliveira et al. (de Oliveira et al., 2022). The analysis revealed that curcumin exerts therapeutic effects on various tumor types, including the inhibition of tumor growth (SMD: −3.03; 95% CI: −3.84, −2.21; *p* < 0.00001), reduction in tumor volume (SMD: −7.30; 95% CI: −11.39,-3.21; *p* < 0.00001), and decrease in tumor weight (SMD: −3.96; 95% CI: −6.22, - 1.70; *p* = 0.0006). Furthermore, studies have indicated that curcumin exhibits a greater affinity for PCa cells than for healthy prostate epithelial cells in individuals without the disease, suggesting its potential as a chemopreventive agent against human PCa (Srivastava et al., 2007). However, the existing body of meta-analytical studies on the efficacy of curcumin in PCa remains insufficient. This study, in contrast, revealed significant reductions in both prostate tumor volume and weight, as well as the inhibition of PCa cell growth, following curcumin administration. These effects were attributed to three primary pathways of action. First, curcumin can impede PCa LNCaP cell viability and induce apoptosis (Mukhopadhyay et al., 2001; Nakamura et al., 2002). Curcumin exhibited anti-proliferative properties in LNCaP cells, resulting in a dose-dependent reduction in DNA synthesis efficiency (Mukhopadhyay et al., 2002). Additionally, it downregulated the expression of G1/S-specific cyclin D1, which is frequently overexpressed in various tumor cells and is a crucial target for inhibiting tumor cell proliferation (Bharti et al., 2003; Shankar and Srivastava, 2007; Mahammedi et al., 2016; Yang et al., 2017; Choi et al., 2019; Termini et al., 2020; Ferlay et al.). Curcumin exerted inhibitory effects on the proliferation of PCa cells by downregulating cyclin D1 expression, thereby impeding the transition of tumor cells from the G1 to S phase. Disruption of cell cycle led to apoptosis. In addition, curcumin upregulated Bax, which promotes apoptosis, and downregulated Bcl-2, which inhibits apoptosis in cancer cells. Bcl-2 and Bax expression is closely associated with apoptosis in cancer cells.

In the present study, curcumin exhibited a noteworthy advantage in suppressing PCNA mRNA and MMP2 mRNA in comparison to the control group (*p* < 0.05). PCNA mRNA, a recently discovered probe for assessing the proliferative activity of PCa cells, reflects the extent of cell proliferation (Squires et al., 2003). PCNA mRNA serves as a robust positive marker of the proliferative activity of PCa cells, thereby emerging as a novel determinant for assessing the biological behavior of PCa. Strong positive expression of PCNA mRNA signifies the presence of PCa cells with heightened proliferative activity, elevated malignancy levels, and an unfavorable prognosis, thus establishing a novel criterion for evaluating the biological behavior of PCa (Park et al., 2005; Prusty and Das, 2005). MMP2 mRNA is a member of the matrix metalloproteinases (MMPs), a group of proteins that have been demonstrated to be associated with tumor metastasis (Dos Reis et al., 2009). Additionally, MMPs play a role in tumor growth regulation by maintaining the integrity of cellular pathways (Dorai et al., 2000a). The expression of MMP2 is minimal in benign prostate hyperplasia tissues, whereas in PCa tissues, its expression level correlates with the degree of proliferation. MMP2 is highly expressed in PCa tissues and plays a crucial role in the metastatic progression of prostate tumors (Dorai et al., 2000b). The findings from the cellular assay demonstrated that curcumin could impede the expression of PCNA and MMP2 in PCa cells, thereby highlighting its distinctive merits in PCa management (Hong et al., 2006; Teiten et al., 2011). However, given the limited number of incorporated studies, further rigorous investigations encompassing both the fundamental and clinical realms are imperative to substantiate the efficacy of curcumin in suppressing PCNA mRNA and MMP2 mRNA.

### 4.3 Current status of clinical research and future application potential

Currently, the use of curcumin for PCa treatment relies primarily on preliminary investigations and limited clinical studies, resulting in insufficient evidence. However, few published clinical studies have substantiated the efficacy and safety of curcumin in the treatment of PCa. For instance, Mahammedi conducted a study involving 30 patients with desmoplasma-resistant PCa, administering a combination of docetaxel/prednisone and curcumin. The findings of the study conducted by Choi et al. (2019) revealed that a significant reduction in PSA levels was observed in 59% of the participants, with 14% achieving complete normalization. Additionally, 40% of the patients reported experiencing symptom relief. Furthermore, the study demonstrated that the adverse effects were attributable to the administration of docetaxel rather than curcumin, as the latter exhibited no toxic effects. The trial, which was randomized and double-blinded, encompassed a sample of 97 individuals diagnosed with PCa and indicated a statistically significant decrease in the proportion of patients experiencing PSA progression over a 6-month treatment period in the curcumin group compared with that in the placebo group (10.3% vs 30.2%, *p* = 0.00259). Additionally, the incidence of adverse events was lower in the curcumin group (15.56%) than that in the placebo group (34.78%). Notably, the Clinical Trial Registry platform (https://beta.clinicaltrials.gov) currently hosts numerous ongoing clinical studies on curcumin for PCa, with some already completed or in progress (e.g., NCT02064673, NCT04731844, NCT03211104, NCT03769766, etc.). Increasing attention on curcumin treatment for PCa indicates its potential for broad clinical applications. Consequently, our future focus will be directed towards closely monitoring clinical studies on curcumin for PCa, aiming to gather additional clinical evidence.

### 4.4 Factors affecting curcumin treatment of PCa

Several clinical studies have demonstrated that curcumin dosage plays a significant role in its effectiveness (de Waure et al., 2023). High doses of curcumin, such as 3.6 g/d for 6 months or 8 g/d for 28 days, have been shown to effectively reduce tumor volume and enhance survival rates (Kuriakose et al., 2016; Santosa et al., 2022). These findings are consistent with those of the present study. Furthermore, studies have indicated that the physicochemical properties of curcumin influence its efficacy. Notably, curcumin exhibits poor water solubility and low systemic bioavailability of approximately 0.47% when administered orally (Mirzaei et al., 2017). Encapsulation of curcumin within polymeric nanocarriers has been suggested as a potential solution to improve its physicochemical properties (Klippstein et al., 2015). Polymer nanocapsules, which are characterized by vesicular nanostructures encompassing an oily core encased by a polymer wall, have been extensively studied to enhance curcumin stability, augment its apparent water solubility, improve its bioavailability, and enhance its *in vivo* anti-inflammatory (Asadirad et al., 2022), neuroprotective (Hoppe et al., 2013), and antitumor effects (Zanotto-Filho et al., 2013). This will significantly enhance the advancement of curcumin-based pharmaceuticals and clinical utilization of curcumin. The findings of this study indicate that curcumin has a more pronounced effect on PC-3 cells than on LNCaP cells. Currently, there is a lack of evidence elucidating the underlying reasons, which is potentially attributable to the limited number of LNCaP cells included in this study (only two studies). Given the limited number of included studies, further research is warranted to validate the effects of curcumin dosage, treatment duration, administration method, PCa cell type, and other relevant factors on its efficacy.

### 4.5 Strengths and limitations of this study

Strengths of this study: This study is the inaugural systematic review of animal studies investigating the effects of curcumin on PCa. The evidence derived from these studies holds immense potential for evaluating the effectiveness and possible clinical applications of curcumin in cancer treatment. Consequently, this evidence can serve as a foundation for the design and implementation of clinical trials as well as the advancement of novel therapeutic interventions.

Limitations of this study: The inability to conduct a meta-analysis of the clinical studies was attributed to the limited number of available trials. This limitation was further compounded by the absence of standardized protocols in the included animal trials, resulting in substantial heterogeneity in terms of study design, disease staging, control group, dose, and regimen. Meta-analysis of animal trials may exhibit even greater heterogeneity than clinical studies (Hooijmans et al., 2014b). Consequently, numerous meta-analyses on animal tests have encountered challenges in addressing this issue (de Oliveira et al., 2022; Luís et al., 2023; Dan et al., 2024). Subgroup analyses have been commonly performed to address heterogeneity. However, owing to the limited number of articles within each subgroup, we conducted a subgroup analysis solely on dosage, precluding the examination of factors, such as the mode of administration, treatment duration, and treatment frequency. The findings from the subgroup analyses indicated that dosage exerted an impact on the results, albeit without a substantial reduction in heterogeneity. The meta-analysis conducted by e Oliveira TV et al. (de Oliveira et al., 2022) examined the impact of curcumin’s mode of administration, dose, and treatment duration on the outcomes reported in various articles. The findings revealed that these factors did not significantly influence the results and there was no substantial reduction in heterogeneity. These findings are consistent with the results of this study. Furthermore, a significant number of experimental studies lack comprehensive reporting of randomization methods, allocation concealment, and randomization of outcome assessments, thereby leading to an indeterminate risk of selection, implementation, and measurement biases. Consequently, there is a pressing need to enhance the methodological rigor of animal testing, given that the findings of such studies frequently inform clinical applications. Petersen et al. (Petersen et al., 2016). Discussed the limitation of interpreting preclinical studies, arguing that this increases the difficulty of evaluating the results of preclinical studies owing to differences in pharmacokinetics and pharmacodynamics between humans and animals. He recommends the creation of consensus guidelines for evaluating the conduct and reporting of preclinical research findings. Meta-analyses of animal experiments should be referenced in the future, which will help improve the quality of evidence in animal experiments.

## 5 Conclusion

Determining the underlying factors contributing to the heterogeneity in animal studies remains challenging because of the substantial variations observed in experimental settings. Nonetheless, animal studies are imperative to unravel the etiology of these diseases and evaluate the safety and effectiveness of therapeutic interventions. In the present investigation, we examined the effects of curcumin in preclinical trials targeting PCa. The findings revealed a favorable outcome associated with curcumin administration in PCa treatment, with dosage potentially influencing its efficacy. Notwithstanding the limited number of incorporated studies and the substantial variability in the outcomes, this meta-analysis methodically and impartially measured the impact of curcumin in the management of PCa, thereby offering significant insights for clinical investigations. Consequently, additional double-blind, placebo-controlled, randomized clinical trials are required to assess the efficacy of curcumin in humans.

## Data Availability

The raw data supporting the conclusion of this article will be made available by the authors, without undue reservation.

## References

[B1] Asadirad A. Nashibi R. Khodadadi A. Ghadiri A. A. Sadeghi M. Aminian A. (2022). Antiinflammatory potential of nano-curcumin as an alternative therapeutic agent for the treatment of mild-to-moderate hospitalized COVID-19 patients in a placebo-controlled clinical trial. Phytother. Res. 36 (2), 1023–1031. 10.1002/ptr.7375 35040210

[B2] Bai Y. Li M. Geng D. Liu S. Chen Y. Li S. (2023). Polyphyllins in cancer therapy: a systematic review and meta-analysis of animal studies. Phytomedicine 121, 155096. 10.1016/j.phymed.2023.155096 37769554

[B3] Barve A. Khor T. O. Hao X. Keum Y. S. Yang C. S. Reddy B. (2008). Murine prostate cancer inhibition by dietary phytochemicals--curcumin and phenyethylisothiocyanate. Pharm. Res. 25 (9), 2181–2189. 10.1007/s11095-008-9574-7 18437538 PMC3465714

[B4] Bharti A. C. Donato N. Singh S. Aggarwal B. B. (2003). Curcumin (diferuloylmethane) down-regulates the constitutive activation of nuclear factor-kappa B and IkappaBalpha kinase in human multiple myeloma cells, leading to suppression of proliferation and induction of apoptosis. Blood 101 (3), 1053–1062. 10.1182/blood-2002-05-1320 12393461

[B5] Cao H. Yu H. Feng Y. Chen L. Liang F. (2017). Curcumin inhibits prostate cancer by targeting PGK1 in the FOXD3/miR-143 axis. Cancer Chemother. Pharmacol. 79 (5), 985–994. 10.1007/s00280-017-3301-1 28391351

[B6] Cheng Y. Yang Y. Wu Y. Wang W. Xiao L. Zhang Y. (2020). The curcumin derivative, H10, suppresses hormone-dependent prostate cancer by inhibiting 17β-hydroxysteroid dehydrogenase type 3. Front. Pharmacol. 11, 637. 10.3389/fphar.2020.00637 32457626 PMC7227374

[B7] Choi Y. H. Han D. H. Kim S. W. Kim M. J. Sung H. H. Jeon H. G. (2019). A randomized, double-blind, placebo-controlled trial to evaluate the role of curcumin in prostate cancer patients with intermittent androgen deprivation. Prostate 79 (6), 614–621. 10.1002/pros.23766 30671976

[B8] Dan L. Hao Y. Song H. Wang T. Li J. He X. (2024). Efficacy and potential mechanisms of the main active ingredients of astragalus mongholicus in animal models of liver fibrosis: a systematic review and meta-analysis. J. Ethnopharmacol. 319 (Pt 1), 117198. 10.1016/j.jep.2023.117198 37722514

[B9] de Oliveira T. V. Stein R. de Andrade D. F. Beck R. C. R. (2022). Preclinical studies of the antitumor effect of curcumin-loaded polymeric nanocapsules: a systematic review and meta-analysis. Phytother. Res. 36 (8), 3202–3214. 10.1002/ptr.7538 35778819

[B10] de Waure C. Bertola C. Baccarini G. Chiavarini M. Mancuso C. (2023). Exploring the contribution of curcumin to cancer therapy: a systematic review of randomized controlled trials. Pharmaceutics 15 (4), 1275. 10.3390/pharmaceutics15041275 37111761 PMC10144810

[B11] Dorai T. Cao Y. C. Dorai B. Buttyan R. Katz A. E. (2001). Therapeutic potential of curcumin in human prostate cancer. III. Curcumin inhibits proliferation, induces apoptosis, and inhibits angiogenesis of LNCaP prostate cancer cells *in vivo* . Prostate 47 (4), 293–303. 10.1002/pros.1074 11398177

[B12] Dorai T. Gehani N. Katz A. (2000a). Therapeutic potential of curcumin in human prostate cancer-I. curcumin induces apoptosis in both androgen-dependent and androgen-independent prostate cancer cells. Prostate Cancer Prostatic Dis. 3 (2), 84–93. 10.1038/sj.pcan.4500399 12497104

[B13] Dorai T. Gehani N. Katz A. (2000b). Therapeutic potential of curcumin in human prostate cancer. II. Curcumin inhibits tyrosine kinase activity of epidermal growth factor receptor and depletes the protein. Mol. Urol. 4 (1), 1–6. 10851300

[B14] Dos Reis S. T. Pontes J. Jr Villanova F. E. Borra P. M. d. A. Antunes A. A. Dall'oglio M. F. (2009). Genetic polymorphisms of matrix metalloproteinases: susceptibility and prognostic implications for prostate cancer. J. Urol. 181 (5), 2320–2325. 10.1016/j.juro.2009.01.012 19303106

[B15] Ferlay J. Ervik M. Lam F. Global cancer observatory: cancer today. France: International Agency for Research on CancerLyon.

[B16] Fernández-Martínez A. B. Bajo A. M. Valdehita A. Isabel Arenas M. Sánchez-Chapado M. Carmena M. J. (2009). Multifunctional role of VIP in prostate cancer progression in a xenograft model: suppression by curcumin and COX-2 inhibitor NS-398. Peptides 30 (12), 2357–2364. 10.1016/j.peptides.2009.09.018 19772879

[B17] Hejazi J. Rastmanesh R. Taleban F. A. Molana S. H. Hejazi E. Ehtejab G. (2016). Effect of curcumin supplementation during radiotherapy on oxidative status of patients with prostate cancer: a double blinded, randomized, placebo-controlled study. Nutr. Cancer 68 (1), 77–85. 10.1080/01635581.2016.1115527 26771294

[B18] Hewlings S. J. Kalman D. S. (2017). Curcumin: a review of its' effects on human Health. foods 6, 92. 10.3390/foods6100092 29065496 PMC5664031

[B19] Higgins J. P. Thompson S. G. (2002). Quantifying heterogeneity in a meta-analysis. Stat. Med. 21 (11), 1539–1558. 10.1002/sim.1186 12111919

[B20] Hong J. H. Ahn K. S. Bae E. Jeon S. S. Choi H. Y. (2006). The effects of curcumin on the invasiveness of prostate cancer *in vitro* and *in vivo* . Prostate Cancer Prostatic Dis. 9 (2), 147–152. 10.1038/sj.pcan.4500856 16389264

[B21] Hooijmans C. R. Rovers M. M. de Vries R. B. Leenaars M. Ritskes-Hoitinga M. Langendam M. W. (2014a). SYRCLE's risk of bias tool for animal studies. BMC Med. Res. Methodol. 14, 43. 10.1186/1471-2288-14-43 24667063 PMC4230647

[B22] Hooijmans C. R. Rovers M. M. de Vries R. B. Leenaars M. Ritskes-Hoitinga M. Langendam M. W. (2014b). SYRCLE's risk of bias tool for animal studies. BMC Med. Res. Methodol. 14, 43. 10.1186/1471-2288-14-43 24667063 PMC4230647

[B23] Hoppe J. B. Coradini K. Frozza R. L. Oliveira C. M. Meneghetti A. B. Bernardi A. (2013). Free and nanoencapsulated curcumin suppress β-amyloid-induced cognitive impairments in rats: involvement of BDNF and Akt/GSK-3β signaling pathway. Neurobiol. Learn Mem. 106, 134–144. 10.1016/j.nlm.2013.08.001 23954730

[B24] Huang H. Chen X. Li D. He Y. Li Y. Du Z. (2015). Combination of α-tomatine and curcumin inhibits growth and induces apoptosis in human prostate cancer cells. PLoS One 10 (12), e0144293. 10.1371/journal.pone.0144293 26630272 PMC4668011

[B25] Ide H. Tokiwa S. Sakamaki K. Nishio K. Isotani S. Muto S. (2010). Combined inhibitory effects of soy isoflavones and curcumin on the production of prostate-specific antigen. Prostate 70 (10), 1127–1133. 10.1002/pros.21147 20503397

[B26] Khor T. O. Keum Y. S. Lin W. Kim J. H. Hu R. Shen G. (2006). Combined inhibitory effects of curcumin and phenethyl isothiocyanate on the growth of human PC-3 prostate xenografts in immunodeficient mice. Cancer Res. 66 (2), 613–621. 10.1158/0008-5472.CAN-05-2708 16423986

[B27] Klippstein R. Wang J. T. El-Gogary R. I. Bai J. Mustafa F. Rubio N. (2015). Passively targeted curcumin-loaded PEGylated PLGA nanocapsules for colon cancer therapy *in vivo* . small 11 (36), 4704–4722. 10.1002/smll.201403799 26140363 PMC4660879

[B28] Kuriakose M. A. Ramdas K. Dey B. Iyer S. Rajan G. Elango K. K. (2016). A randomized double-blind placebo-controlled phase IIB trial of curcumin in oral leukoplakia. Cancer Prev. Res. ( Phila) 9 (8), 683–691. 10.1158/1940-6207.CAPR-15-0390 27267893

[B29] Li H. F. Chen F. M. Shi J. Q. (2013). Tumor-suppressive effects of toughening moiety-curcumin monolipids enhanced on prostate cancer. Chin. J. Exp. Surg. 30 (12), 2493–2495. 10.3760/cma.j.issn.1001-9030.2013.12.004

[B30] Limin L. Kong X. Chang X. (2007). Experimental study on the effect of curcumin on prostate cancer PC-3M graft tumors. Chin. J. Gerontology 27 (1), 45–47. 10.3969/j.issn.1005-9202.2007.01.019

[B31] Luís Â. Marcelino H. Domingues F. Pereira L. Cascalheira J. F. (2023). Therapeutic potential of resveratrol for glioma: a systematic review and meta- analysis of animal model studies. Int. J. Mol. Sci. 24 (23), 16597. 10.3390/ijms242316597 38068922 PMC10706392

[B32] Mahammedi H. Planchat E. Pouget M. Durando X. Curé H. Guy L. (2016). The new combination docetaxel, prednisone and curcumin in patients with castration-resistant prostate cancer: a pilot phase II study. Oncology 90 (2), 69–78. 10.1159/000441148 26771576

[B33] Mao J. Xiong X. Gong H. (2019). Effects of curcumin on tumor growth and immune function in prostate cancer-bearing mice. Chin. J. Male Sci. 25 (7), 590–594. 10.13263/j.cnki.nja.2019.07.003 32223098

[B34] Mirzaei H. Shakeri A. Rashidi B. Jalili A. Banikazemi Z. Sahebkar A. (2017). Phytosomal curcumin: a review of pharmacokinetic, experimental and clinical studies. Biomed. Pharmacother. 85, 102–112. 10.1016/j.biopha.2016.11.098 27930973

[B35] Mukhopadhyay A. Banerjee S. Stafford L. J. Xia C. Liu M. Aggarwal B. B. (2002). Curcumin-induced suppression of cell proliferation correlates with down-regulation of cyclin D1 expression and CDK4-mediated retinoblastoma protein phosphorylation. oncogene 21 (57), 8852–8861. 10.1038/sj.onc.1206048 12483537

[B36] Mukhopadhyay A. Bueso-Ramos C. Chatterjee D. Pantazis P. Aggarwal B. B. (2001). Curcumin downregulates cell survival mechanisms in human prostate cancer cell lines. Oncogene 20 (52), 7597–7609. 10.1038/sj.onc.1204997 11753638

[B37] Nakamura K. Yasunaga Y. Segawa T. Ko D. Moul J. W. Srivastava S. (2002). Curcumin down-regulates AR gene expression and activation in prostate cancer cell lines. Int. J. Oncol. 21 (4), 825–830. 10.3892/ijo.21.4.825 12239622

[B38] National Health Commission of the People's Republic of China (2019). Chinese guidelines for diagnosis and treatment of prostate cancer 2018 (English version). Chin. J. Cancer Res. 31 (1), 67–83. 10.21147/j.issn.1000-9604.2019.01.04 31156298 PMC6513740

[B39] Park C. H. Lee J. H. Yang C. H. (2005). Curcumin derivatives inhibit the formation of Jun-Fos-DNA complex independently of their conserved cysteine residues. J. Biochem. Mol. Biol. 38 (4), 474–480. 10.5483/bmbrep.2005.38.4.474 16053715

[B40] Petersen G. H. Alzghari S. K. Chee W. Sankari S. S. La-Beck N. M. (2016). Meta-analysis of clinical and preclinical studies comparing the anticancer efficacy of liposomal versus conventional non-liposomal doxorubicin. J. Control Release 232, 255–264. 10.1016/j.jconrel.2016.04.028 27108612

[B41] Prusty B. K. Das B. C. (2005). Constitutive activation of transcription factor AP-1 in cervical cancer and suppression of human papillomavirus (HPV) transcription and AP-1 activity in HeLa cells by curcumin. Int. J. Cancer 113 (6), 951–960. 10.1002/ijc.20668 15514944

[B42] Qi R. (2009). Effects of curcumin combined with paclitaxel on proliferation and invasion of prostate cancer PC-3 cells *in vitro* and *in vivo* . Shandong: Qingdao University. 10.7666/d.y1469102

[B43] Qi R. Yu X. Zhao H. (2015). Inhibitory effect of curcumin on the migration of PC-3 cells in prostate cancer and its effect on the expression of MMP2 *in vivo* and *in vitro* . China J. Basic Chin. Med. 21 (11), 1398–1400.

[B44] Roy L. Zhang A. Zhang J. (2016). Study on the effect of curcumin on prostate cancer PC-3 nude mice transplanted tumors, 353–354.

[B45] Santosa D. Suharti C. Riwanto I. Dharmana E. Pangarsa E. A. Setiawan B. (2022). Curcumin as adjuvant therapy to improve remission in myeloma patients: a pilot randomized clinical trial. Casp. J. Intern Med. 13 (2), 375–384. 10.22088/cjim.13.2.9 PMC9301229 35919637

[B46] Sena E. S. Currie G. L. McCann S. K. Macleod M. R. Howells D. W. (2014). Systematic reviews and meta-analysis of preclinical studies: why perform them and how to appraise them critically. J. Cereb. Blood Flow. Metab. 34 (5), 737–742. 10.1038/jcbfm.2014.28 24549183 PMC4013765

[B47] Shankar S. Srivastava R. K. (2007). Involvement of Bcl-2 family members, phosphatidylinositol 3'-kinase/AKT and mitochondrial p53 in curcumin (diferulolylmethane)-induced apoptosis in prostate cancer. Int. J. Oncol. 30 (4), 905–918. 10.3892/ijo.30.4.905 17332930

[B48] Singh D. Gupta M. Sarwat M. Siddique H. R. (2022). Apigenin in cancer prevention and therapy: a systematic review and meta-analysis of animal models. Crit. Rev. Oncol. Hematol. 176, 103751. 10.1016/j.critrevonc.2022.103751 35752426

[B49] Spanagel R. . (2022). Ten points to improve reproducibility and translation of animal research. Front. Behav. Neurosci. 16, 869511. 10.3389/fnbeh.2022.869511 35530730 PMC9070052

[B50] Squires M. S. Hudson E. A. Howells L. Sale S. Houghton C. E. Jones J. L. (2003). Relevance of mitogen activated protein kinase (MAPK) and phosphotidylinositol-3-kinase/protein kinase B (PI3K/PKB) pathways to induction of apoptosis by curcumin in breast cells. Biochem. Pharmacol. 65 (3), 361–376. 10.1016/s0006-2952(02)01517-4 12527329

[B51] Srivastava R. K. Chen Q. Siddiqui I. Sarva K. Shankar S. (2007). Linkage of curcumin-induced cell cycle arrest and apoptosis by cyclin-dependent kinase inhibitor p21(/WAF1/CIP1). Cell Cycle 6 (23), 2953–2961. 10.4161/cc.6.23.4951 18156803

[B52] Sung H. Ferlay J. Siegel R. L. Laversanne M. Soerjomataram I. Jemal A. (2021). Global cancer statistics 2020: GLOBOCAN estimates of incidence and mortality worldwide for 36 cancers in 185 countries. CA Cancer J. Clin. 71 (3), 209–249. 10.3322/caac.21660 33538338

[B53] Teiten M. H. Gaascht F. Cronauer M. Henry E. Dicato M. Diederich M. (2011). Anti-proliferative potential of curcumin in androgen-dependent prostate cancer cells occurs through modulation of the Wingless signaling pathway. Int. J. Oncol. 38 (3), 603–611. 10.3892/ijo.2011.905 21240460

[B54] Termini D. Den Hartogh D. J. Jaglanian A. Tsiani E. (2020). Curcumin against prostate cancer: current evidence. Biomolecules 10 (11), 1536. 10.3390/biom10111536 33182828 PMC7696488

[B55] Vesterinen H. M. Sena E. S. Egan K. J. Hirst T. C. Churolov L. Currie G. L. (2014). Meta-analysis of data from animal studies: a practical guide. J. Neurosci. Methods 221, 92–102. 10.1016/j.jneumeth.2013.09.010 24099992

[B56] Yang J. Ning J. Peng L. He D. (2015). Effect of curcumin on Bcl-2 and Bax expression in nude mice prostate cancer. Int. J. Clin. Exp. Pathol. 8 (8), 9272–9278. 26464676 PMC4583908

[B57] Yang J. Wang C. Zhang Z. Chen X. Jia Y. Wang B. (2017). Curcumin inhibits the survival and metastasis of prostate cancer cells via the Notch-1 signaling pathway. apmis 125 (2), 134–140. 10.1111/apm.12650 28120490

[B58] Yang L. Zhang L. Y. Chen W. W. Kong F. Zhang P. J. Hu X. Y. (2005). Inhibition of the expression of prostate specific antigen by curcumin. Yao Xue Xue Bao 40 (9), 800–803. 16342680

[B59] Ye W. (2015). Inhibitory effect of curcumin combined with TfRmAb on human prostate cancer PC-3 cells transplanted tumor in nude mice. Hubei: Huazhong University of Science and Technology. 10.7666/d.D735214

[B60] Zanotto-Filho A. Coradini K. Braganhol E. Schröder R. de Oliveira C. M. Simões-Pires A. (2013). Curcumin-loaded lipid-core nanocapsules as a strategy to improve pharmacological efficacy of curcumin in glioma treatment. Eur. J. Pharm. Biopharm. 83 (2), 156–167. 10.1016/j.ejpb.2012.10.019 23219677

[B61] Zhang A. (2013). Inhibitory effect and mechanism of curcumin on prostate cancer PC-3 cell line transplanted tumor in nude mice. Kunming Medical University.

[B62] Zhao W. Zhou X. Qi G. Guo Y. (2018). Curcumin suppressed the prostate cancer by inhibiting JNK pathways via epigenetic regulation. J. Biochem. Mol. Toxicol. 32 (5), e22049. 10.1002/jbt.22049 29485738

[B63] Zhao H. Yu X. Qi R. (2010). Study on the effect of curcumin and paclitaxel combination on prostate cancer PC-3 transplantation tumor in nude mice. Mod. Biomed. Prog. 10 (5), 823–827.

